# An interpretable artificial intelligence system for detecting risk factors of gastroesophageal variceal bleeding

**DOI:** 10.1038/s41746-022-00729-z

**Published:** 2022-12-19

**Authors:** Jing Wang, Zhengqiang Wang, Mingkai Chen, Yong Xiao, Shi Chen, Lianlian Wu, Liwen Yao, Xiaoda Jiang, Jiao Li, Ming Xu, Mengjuan Lin, Yijie Zhu, Renquan Luo, Chenxia Zhang, Xun Li, Honggang Yu

**Affiliations:** 1 Department of Gastroenterology, Renmin Hospital of Wuhan University, Wuhan, China; 2 Hubei Provincial Clinical Research Center for Digestive Disease Minimally Invasive Incision, Renmin Hospital of Wuhan University, Wuhan, China; 3 Key Laboratory of Hubei Province for Digestive System Disease, Renmin Hospital of Wuhan University, Wuhan, China; 4 Department of Gastroenterology, Wuhan Puren Hospital, Wuhan, China

**Keywords:** Oesophageal diseases, Risk factors

## Abstract

Bleeding risk factors for gastroesophageal varices (GEV) detected by endoscopy in cirrhotic patients determine the prophylactical treatment patients will undergo in the following 2 years. We propose a methodology for measuring the risk factors. We create an artificial intelligence system (ENDOANGEL-GEV) containing six models to segment GEV and to classify the grades (grades 1–3) and red color signs (RC, RC0-RC3) of varices. It also summarizes changes in the above results with region in real time. ENDOANGEL-GEV is trained using 6034 images from 1156 cirrhotic patients across three hospitals (dataset 1) and validated on multicenter datasets with 11009 images from 141 videos (dataset 2) and in a prospective study recruiting 161 cirrhotic patients from Renmin Hospital of Wuhan University (dataset 3). In dataset 1, ENDOANGEL-GEV achieves intersection over union values of 0.8087 for segmenting esophageal varices and 0.8141 for gastric varices. In dataset 2, the system maintains fairly accuracy across images from three hospitals. In dataset 3, ENDOANGEL-GEV surpasses attended endoscopists in detecting RC of GEV and classifying grades (*p* < 0.001). When ranking the risk of patients combined with the Child‒Pugh score, ENDOANGEL-GEV outperforms endoscopists for esophageal varices (*p* < 0.001) and shows comparable performance for gastric varices (*p* = 0.152). Compared with endoscopists, ENDOANGEL-GEV may help 12.31% (16/130) more patients receive the right intervention. We establish an interpretable system for the endoscopic diagnosis and risk stratification of GEV. It will assist in detecting the first bleeding risk factors accurately and expanding the scope of quantitative measurement of diseases.

## Introduction

Decompensated cirrhosis is defined in terms of development of ascites, variceal hemorrhage, or hepatic encephalopathy^1^. Gastroesophageal varices (GEV) are severe complications of cirrhosis present in 85% of decompensated cirrhotic patients, and consequent variceal hemorrhage is life-threatening^2,3^. Although noninvasive methods have been adopted to exclude patients who are unlikely to develop GEV, endoscopy is the gold standard for diagnosing GEV and predicting the risk of hemorrhage within 2 years^4^. Patients diagnosed with cirrhosis should undergo endoscopy to detect varices and rank the risk of variceal bleeding. Endoscopic risk rank determines different treatment recommendations for primary prophylaxis in the following 1–2 years^5^. Cirrhotic patients with grade 1 varices and red color signs (RC)/Child‒Pugh C or grade 2–3 varices should receive prophylactic treatments according to guidelines^6,7^. Several studies also confirmed the significance of endoscopic risk factors for predicting variceal hemorrhage, one of the manifestations of decompensated cirrhosis^8,9^.

However, the endoscopic description of risk factors is subject to operator dependence. There is a low consistency among endoscopists on the grade, RC, and size of GEV^10,11^. Incorrect diagnoses by endoscopists come at the expense of the patients’ security and medical costs. Nonselective beta-blockers or variceal band ligation should be primary prophylaxis for high-risk patients. If patients with high-risk varices were missed, the rupture rate of varices is ~15% per year, and the mortality is up to 25% in 6 weeks^8,12^. Low-risk patients should be screened every 2 years. If low-risk patients are misdiagnosed as high-risk patients, they might experience side effects but not benefit from prophylaxis, including postoperative bleeding and bradycardia, etc. What is worse, similar research indicated that subjectivity is inherent in humans, and training may only poorly improve it^13^. A clinical method that provides a quantitative and accurate assessment of endoscopic risk factors is urgently needed.

With the significant advances of artificial intelligence (AI) in endoscopy, AI has made up for the shortcomings of endoscopists and normalized the diagnoses made by endoscopists^13,14^. AI was successfully used to help endoscopists detect colorectal adenomas during colonoscopy, and to reduce blind spots during esophagogastroduodenoscopy, etc. Several studies used computed tomography to diagnose high bleeding risk esophageal varices (EV)^15–18^. However, these studies were limited in small sample size, retrospective design, and low area under the curves. More accurate methods, and larger multicenter validations are still needed. Deep convolutional neural networks (DCNN) were also used in endoscopy to detect EV and gastric varices (GV)^10,19^. These systems conclude whether varices appear in an image or video, but don’t point out where the lesion is. A growing number of people believe that the “black box” features of AI attenuated its reliability^20^. Although algorithms perform excellently in a broad spectrum of diseases including varices, why they make such decisions is difficult to interpret^21^. Interpretable AI is attracting much interest in medicine, but most of the attempts so far, such as Shapley values, were for developers, not end-users. The Shapley value of features on the image is calculated by retraining models after the removal the features. But its long computing time does not allow Shapley value to be displayed to users in time^22,23^. To address these limitations, we developed an AI system that is explainable for both developers and end-users to delineate GEV.

In this study, we provide visualized, objective, and quantitative deep learning measurements to predict the risk factors for GEV hemorrhage. The system is designed to identify patients with high bleeding risk by segmenting the varices and RC on the varices and further classifying the grade (size) of varices and the density and distribution of RC. The changes and accumulated percentages of grade(size) and RC during esophagogastroduodenoscopy would be calculated to visualize the prediction of the system. The system shows robust performance in the observational study. The system will increase the effectiveness of interventions tailored to the risk of hemorrhage, improve health outcomes of cirrhotic patients and reduce spending on healthcare.

## Results

### System construction

The system consists of the main models for GEV diagnosis and risk factors detection, and supportive models for unqualified images deletion. Main models include EV segmentation model(model 1), RC segmentation model(for both EV and GV, model 2), RC classification model (for EV, model 3)and grade classification model(for EV, model 4), GV segmentation (model 5), size classification model for GV (model 6).

### Demographics

**Fig. 1:**
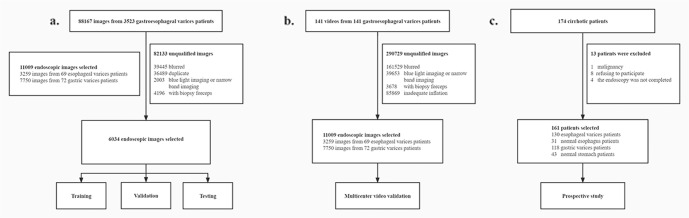
The flowchart of research. **a** Data processing of dataset 1(Training, validation and testing). **b** Data processing of dataset 2(Validation dataset). **c** Data processing of dataset 3(Prospective study).

From July 1st, 2020, to April 30th, 2021, 174 cirrhotic patients undergoing endoscopic screening for varices were eligible for inclusion in the study. Thirteen patients were excluded because of malignancy (*n* = 1), refusal to participate in this study (*n* = 8), or incomplete endoscopy (*n* = 4). Therefore, 161 patients were analyzed in this research (117 men, 44 women; mean age 57.41 years, range 32–79 years). The demographic data are summarized in Table 1. The flowchart of the dataset preparation is shown in Fig. 1.

**Table 1:** Clinical characteristics of patients.

Dataset characteristics	Dataset 1(training and validation)	Dataset 1(Testing dataset)	Dataset 2(Validation dataset)	Dataset 3(Prospective study)
Age, years, (Range)	59.00 (28–80)	56.63 (24–75)	58.32 (29–84)	57.41 (32–79)
Sex, (%)				
Men	736 (72.16)	88 (64.71)	47 (67.14)	117 (72.96)
Women	284 (27.84)	48 (35.29)	23 (32.86)	44 (27.04)
Etiology, (%)				
HBV	540 (52.94)	76 (55.88)	36 (51.43)	79 (49.06)
HCV	100 (9.80)	8 (5.88)	6 (8.57)	16 (9.93)
Alcoholic	120 (11.76)	18 (13.24)	8 (11.43)	22 (13.66)
Schistosomiasis	124 (12.16)	10 (7.35)	9 (12.86)	21 (13.04)
Other	136 (13.33)	24 (17.65)	11 (15.71)	23 (14.28)
Child-Pugh, (%)				
A	252 (24.71)	18 (13.24)	21 (30.00)	73 (45.34)
B	564 (55.29)	92 (67.65)	33 (47.14)	73 (45.34)
C	144 (14.12)	26 (19.12)	16 (22.86)	15 (9.31)

*HBV* Hepatitis B virus, *HCV* Hepatitis C virus.

### The performance of ENDOANGEL-GEV on dataset 1(Testing dataset)

**Fig. 2:**
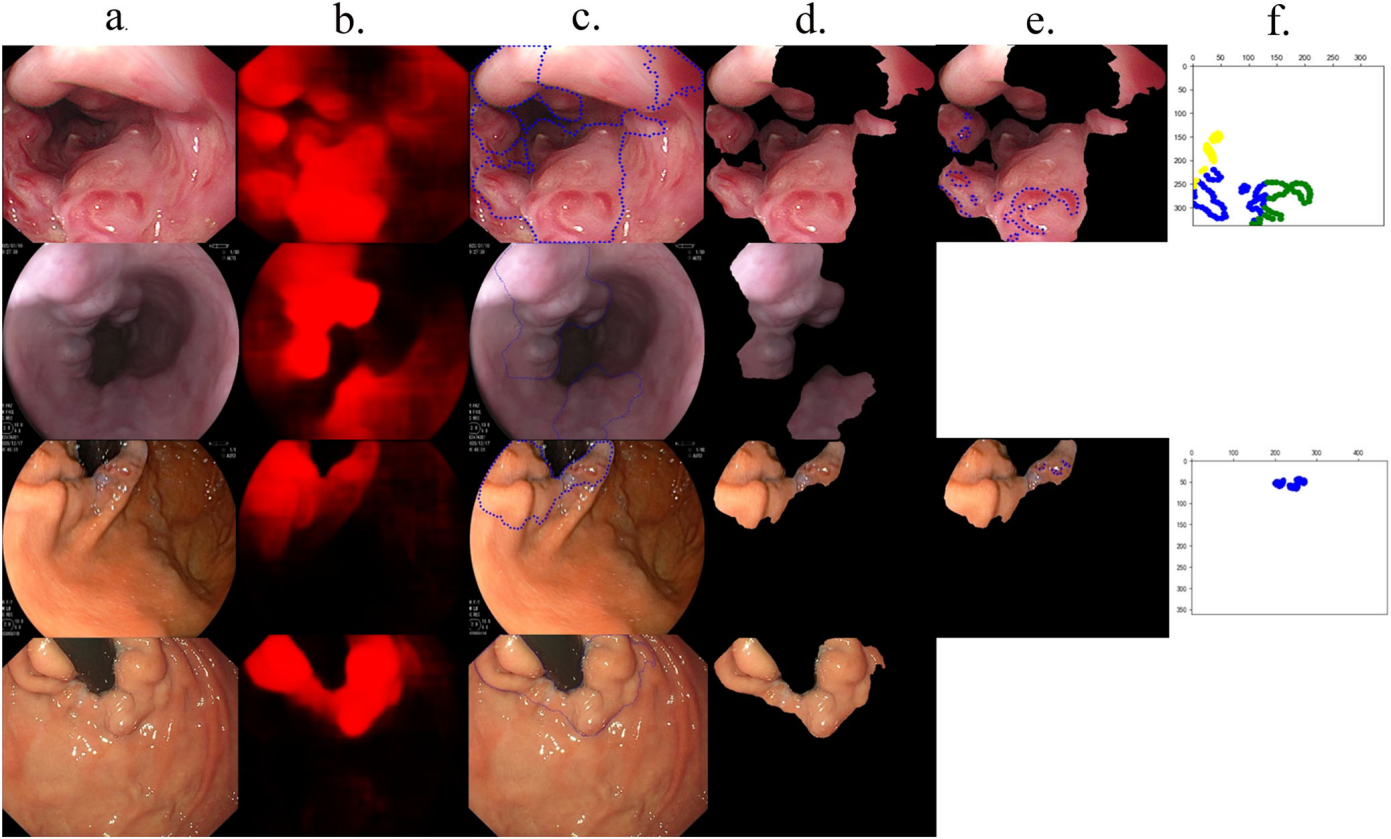
Representative images of how the system processes images. **a** The first column shows the original endoscopic images. **b** The second column shows model 1 (EV segmentation) and model 5 (GV segmentation) generate a probability map (heat map) for each image internally. The brighter the color in the image, the higher the likelihood the region is varices. A cut-off value of 0.4 was chosen to delineate the regions of GEV. **c** The delineation of varices on the endoscopic images is shown in this column. **d** Nonvariceal regions were deleted in this column. **e** RC were delineated on the varices in this column. A cut-off value of 0.5 was chosen to delineate RC. **f** Density-based spatial clustering of applications with noise model divides the red color signs into different groups, and groups were represented as different colors.

The system’s performance is summarized in Table 2, and Supplementary Tables 2–3. Representative images of the system are shown in Fig. 2. Model 1(EV segmentation model) detected EV with a sensitivity of 93.49% (95% confidence interval (CI), 92.04–94.74%) per varix (1351 varices). Model 1 delineated the outlines of EV with a mean intersection over union (mIoU) of 0.8087 (95% CI, 0.7968–0.8206). Model 2 (RC segmentation model) achieved an accuracy of 97.80% (95% CI, 96.10–98.90%) for detecting RC of EV. Model 3 (RC classification model) reached an accuracy of 94.40% (95% CI, 92.01–96.25%) for the classification of RC with accuracies of 89.24% (95% CI, 80.68–94.44%), 91.67% (95% CI, 83.04–96.30%), and 95.83% (95% CI, 89.07–98.65%) for RC1, RC2, and RC3, respectively. Model 4 (grade classification model) correctly classified 93.00% (95% CI, 90.40–95.08%) of images for the grade of EV. Model 4 reached accuracies of 90.00% (95% CI, 83.05–94.68%), 93.19% (95% CI, 89.87–95.68%) and 98.27% (95% CI, 90.76–99.96%) for classifying grade 1, 2 and grade 3, respectively.

**Table 2:** Diagnostic value of model 1 and model 5 for the detection of varices across all datasets.

	Precision, %(95% CI)	Recall, %(95% CI)	mIoU(95% CI)	mIoU > 0.5, %(95% CI)	mIoU > 0.6, %(95% CI)	Sensitivity, %(95% CI) (Per varix)	Sensitivity, %(95% CI) (Per image)
*EV*							
Dataset 1 (Testing dataset)	86.12 (85.43, 86.81)	87.06 (85.94, 88.18)	0.8087 (0.7968, 0.8206)	90.60 (87.70, 93.01)	85.80 (82.43, 88.74)	93.49 (92.04, 94.74)	100.00 (99.26, 100.00)
Dataset 2(Validation dataset)	87.09 (86.55, 87.61)	91.66 (90.93, 92.38)	0.8890 (0.8811, 0.8970)	92.89 (91.50, 94.12)	90.44 (88.86, 91.86)	90.79 (89.55, 91.91)	92.08 (90.54, 93.40)
Dataset 3(Prospective study)	90.07 (89.51, 90.63)	89.98 (89.04, 90.92)	0.8494 (0.8398, 0.8590)	91.60 (90.26, 92.81)	88.82 (87.32, 90.20)	91.63 (91.56, 92.52)	99.84 (99.50, 99.96)
*GV*							
Dataset 1 (Testing dataset)	92.35 (91.68, 93.02)	89.94 (88.97, 90.91)	0.8141 (0.8087, 0.8195)	88.36 (85.38, 90.92)	81.09 (77.56, 84.28)	95.93 (94.33, 97.18)	100.00 (99.33, 100.00)
Dataset 2(Validation dataset)	88.67 (87.46, 87.87)	91.04 (90.82, 91.25)	0.8551 (0.8524, 0.9077)	92.33 (90.33, 94.33)	90.93 (88.86, 93.00)	88.34 (87.76, 88.90)	99.72 (99.58, 99.82)
Dataset 3(Prospective study)	90.21 (89.75, 90.66)	91.31 (90.78, 91.84)	0.8734 (86.18, 87.98)	91.21 (89.92, 93.35)	90.43 (89.09, 91.62)	90.94 (89.71, 92.05)	100.00 (99.78, 100.00)

*EV* esophageal varices, *GV* gastric varices, *mIoU* mean intersection over union.

Model 5(GV segmentation model) achieved a sensitivity of 95.93% (95% CI, 94.33–97.18%) per varix (811 varices) for detecting GV and delineated GV with a mIoU of 0.8141 (95% CI, 0.8087–0.8195). Model 3(RC segmentation model) achieved an accuracy of 90.73% (95% CI, 87.99–93.02%), a sensitivity of 90.03% (95% CI, 86.09–92.98%) and a specificity of 91.70% (95% CI, 87.15–94.79%) for detecting RC of GV.

### The performance of ENDOANGEL-GEV on dataset 2(validation dataset)

Model 1(EV segmentation model) achieved a sensitivity of 90.79% (95% CI, 89.55–91.91%) for detecting EV (2402 varices). It delineated EV with a mIoU of 0.8890(95% CI, 0.8811, 0.8970). Model 2 (RC segmentation model) achieved a sensitivity of 99.79% (95% CI, 99.65–100.00%) and, a specificity of 92.54% (95% CI, 91.23–93.66%) for predicting RC of EV. Model 3 (RC classification model) achieved an accuracy of 93.43% (95% CI, 92.51–94.24%) for the classification of RC Model 4 (grade classification model) classified EV grades 1–3 with accuracies of 94.84% (95% CI, 91.62–96.04%), 93.67% (95% CI, 90.57–94.75%), and 93.88% (95% CI, 89.74–96.82%).

Model 5(GV segmentation model) diagnosed GV with a sensitivity of 88.34% (95% CI, 87.76–88.90%) per varix (12383 varices). Model 5 delineated the outlines of GV with a mIoU of 0.8551(95% CI, 0.8524, 0.9077). Model 3(RC segmentation model) achieved a sensitivity of 91.10% (95% CI, 89.85–92.20%) and a specificity of 91.58% (95% CI, 90.80–92.30%) for detecting RC of GV.

### Comparison between ENDOANGEL-GEV and endoscopists on dataset 3(Prospective study)

**Fig. 3:**
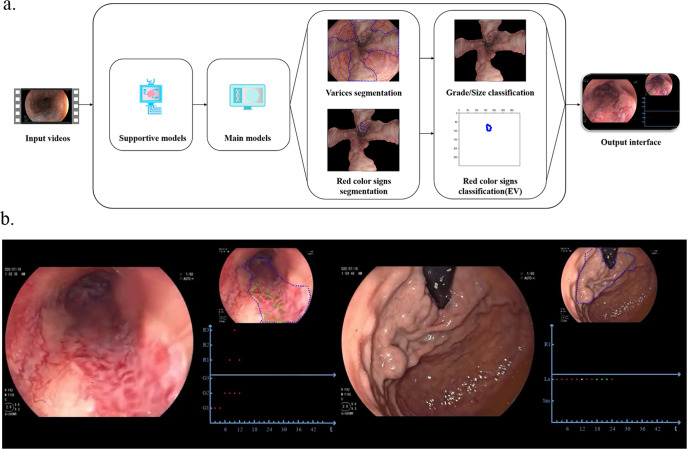
The framework and interface of the system. **a** The framework of the system. Videos are input into the system and are analyzed seven frames per second. Firstly, supportive models filter unqualified images, and qualified images are processed by main models. Model 1, 2, 5 delineate GEV and RC, then model 3, 4, 6 classify grade and RC. All above features are summarized according to time and regions, and accumulated results are shown on the interface. **b** The interface of the system. The predicted results were shown on the upper right of the screen. The summarized results change with time, and the region is listed on the interface’s lower right. G1: grade 1, G2: grade 2, G3: grade 3, R1: RC1, R2:RC2, R3: RC3, La: large, Sm: small, Red spots: greater curvature, green spots: posterior wall, white spots: anterior wall, blue spots, lesser curvature. GEV gastroesophageal varices, RC red color signs.

**Fig. 4:**
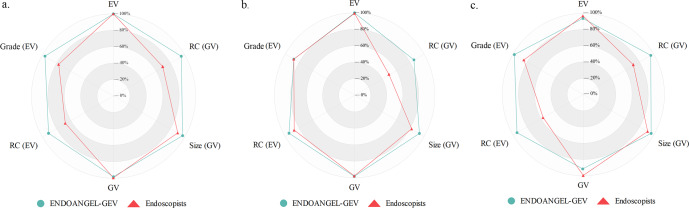
Comparison of the results between ENDOANGEL-GEV and endoscopists on dataset 3(Prospective study). Radar maps illustrate the accuracy (**a**), sensitivity (**b**), and specificity (**c**) on dataset 3. In this figure, grade 1 is regarded as small, and grade 2–3 is seen as large for classifying grade. EV esophageal varices, GV gastric varices, RC red color signs.

The videos in dataset 3 were processed according to Fig. 3. Representative original images and qualified images filtered by supportive models are shown in Supplementary Figs. 1 and 2. Figure 4 presents the diagnostic yields of ENDOANGEL-GEV and endoscopists on dataset 3. The sensitivity of ENDOANGEL-GEV for detecting EV was comparable to that of endoscopists (100.00%, 95% CI 96.44–100.00% vs. 99.23%, 95% CI 95.19–99.96%, *p* = 1.000) The accuracy of ENDOANGEL-GEV for classifying RC was significantly higher than that of endoscopists (94.62%, 95% CI 89.11–97.56% vs. 66.92%, 95% CI 58.44–74.44%, *p* < 0.001). ENDOANGEL-GEV ranked grade better than endoscopists (94.57%, 95CI% 89.14–99.90% vs. 75.97%, 95% CI 67.66–83.05%, *p* < 0.001).

ENDOANGEL-GEV showed comparable performance with endoscopists in detecting GV (97.52%, 95% CI, 93.57–99.25% vs. 98.76%, 95%, CI 95.30–99.95%, *p* = 0.625) The accuracy of ENDOANGEL-GEV in classifying the RC of GV is significantly higher than that of endoscopists. (94.92%, 95% CI 89.26–98.11% vs. 69.49%, 95% CI 60.34–7.63%, *p* < 0.001).

Regarding the risk stratification of EV using endoscopic findings and Child-Pugh score, ENDOANGEL-GEV significantly outperformed endoscopists (97.69%, 95% CI, 93.14–99.51% vs. 85.38%, 95% CI, 78.22–90.52%, *p* < 0.001) (Table 3). ENDOANGEL-GEV and endoscopists showed similar metrics for ranking the risk of GV (95.76%, 95% CI, 90.21–98.43% vs. 85.38%, 95% CI, 78.22–90.52%, *p* = 0.152) (Table 3). More results are shown in Supplementary Tables 4–13 and Supplementary video 1.

**Table 3:** Diagnostic value of ENDOANGEL-GEV and endoscopists for risk stratification of EV and GV on Dataset 3 (Prospective study).

	The number of images	ENDOANGEL-GEV		Endoscopists	
		High risk(%)	Low risk(%)	High risk(%)	Low risk(%)
Gold standard	EV				
	High risk	110 (84.62)	1 (0.76)	104 (80.00)	7 (5.38)
	Low risk	2 (1.54)	17 (13.08)	12 (9.23)	7 (5.38)
	GV				
	High risk	41 (34.75)	2 (1.69)	37 (31.36)	6 (5.08)
	Low risk	3 (2.54)	72 (61.02)	15 (12.71)	60 (50.85)

*EV* esophageal varices, *GV* gastric varices.

The median follow-up was 12.12 months. Six patients experienced rebleeding (3 EV and 3 GV), and none of the patients died during follow-up (Supplementary Table 14). The results of questionnaire on the satisfaction are shown in supplementary fig. 4.

## Discussion

We developed an interpretable, quantitative, expert-level system for detecting the risk factors of first bleeding of GEV in cirrhosis. Our system yielded high predictive accuracy, detailed assessment, and interpretable results, providing a means for further endoscopic exploration of cirrhosis. ENDOANGEL-GEV outperformed expert panel for ranking the risk of bleeding in real world. Moreover, the high consistency between cohorts with high variance in endoscopy brands and the quality of images indicates a substantial degree of generalizability. These new achievements will facilitate the application of explainable AI in medical training and expand the new scope of quantification in medicine.

All cirrhotic patients suspected to have GEV will undergo endoscopy to assess the risk of rupture within 2 years. Misdiagnosis will expose high-risk patients to the risk of bleeding, and low-risk patients will suffer the side effects of unnecessary treatment. Noninvasive tests accurately identify patients without varices but are not recommended to diagnose GEV^24^. Endoscopy is still the gold standard for diagnosing GEV. The presence of varices indicates a higher portal pressure level, but the endoscopic findings are not correlated with a specific portal pressure level. Higher portal pressure, large varices, and RC are positively correlated with variceal bleeding, while there is no close correlation between changes in portal pressure and changes in the endoscopic findings of varices^25,26^. Therefore, the endoscopic findings of varices are relatively independent indicators of variceal bleeding. Classifying the endoscopic findings of GEV is essential to predict the risk of variceal bleeding.

Quantification is a key point in the endoscopic assessment of GEV: the size of varices and the density and distribution of RC are divided into three groups for further measurement. Quantifying lesion features is also challenging for doctors. For example, endoscopists could not classify the bowel preparation into four groups well enough^27^. The thresholds between groups are unclear and qualitative, which leads to high inconsistency between endoscopists^28^. These refined tasks are also challenging for DCNN, which perform classification based on the whole image. In our previous study, the DCNN model achieved an accuracy of 63.44% (95% CI, 58.30–68.30%) for ranking the grade of varices^10^. Therefore, we introduced fully convolutional networks (FCNs) to help endoscopists perform more delicate tasks such as quantifying complex features and detecting small targets^29^. Instead of semantic segmentation, this study extended the application of FCN to disease classification, presenting the results directly on endoscopic images. We linked FCN to DCNN and achieved a significantly higher accuracy of 93.00% (95% CI, 90.40–95.08%) for classifying grade. We adopted a slightly different method by linking the FCN to DBSCAN to classify RC density and distribution, generating intuitive density and distribution maps for endoscopists. Erosion (25/31, 80.64%), ulcers (3/31, 9.67%), and other mucosal injuries (3/31, 9.67%) on the esophagus and stomach are usually mistaken for RC by endoscopists (Supplementary fig. 4). In comparison, ENDOANGEL-GEV could detect real RCs, which appear as dark red spots under the mucosa. Erosion is also the leading cause (3/4, 75%) of false positives of ENDOANGEL-GEV.

Explainability has been accompanying AI in medicine. The Federal Trade Commission reported using AI and algorithms, mentioning that models should explain their decision to the consumer. If models are used to assign risk scores to consumers, they should disclose and rank the factors that affected the results^30^. An article published in Nature Medicine also pointed out, “AI in medicine must be explainable”^31^. According to our interview and related articles, end-users also need an explainable interface to build trust^32^. Compared with our previously published study, this system will help end-users to understand how it makes its conclusions^33^. DCNN models conceal the features supporting their predictions, preventing people from exploring or optimizing them. Compared with DCNN models, ENDOANGEL-GEV estimates every pixel on the image, providing an intuitionistic prediction of the ill region. Interpretable and direct presentation exposes the model’s logic, directly paving the way for fixing mistakes, explaining to end-users, and training. The questionnaire results also indicated that explainable AI systems are more likely to be accepted by endoscopists (Supplementary Fig. 4).

Articles analyzing the risk factors for first variceal bleeding vary considerably in the level of detail for measuring endoscopic findings^7,34^. One of the reasons may be that it is difficult to unify the criteria for classifying the findings. GV are described in less minor detail than EV because the prevalence of GV is lower than that of EV, and GV bleeding is less correlated with portal vein pressure than EV^35,36^. In our study, endoscopists showed a higher false-positive rate in both RC and grade, while ENDOANGEL-GEV maintained high specificity and remained fairly sensitive. ENDOANGEL-GEV outperformed endoscopists in classifying high-risk patients and low-risk patients. The system will help more patients receive prophylactic therapy and reduce health care waste by freeing 52.63% (10/19) more low-risk patients from unnecessary treatment. In summary, ENDOANGEL-GEV will resolve the inconsistencies and assess infrequent features accurately, contributing to a more detailed clinical analysis.

Lesions in the digestive tract can be divided into solitary lesions (polys, cancers, etc.) and diffuse lesions (inflammatory bowel disease, gastritis, etc.). Previous research mainly provided algorithms more suitable for solitary lesions, such as detecting scattered lesions or diagnosing lesions^37^. However, it is equally important to describe how the lesions change with location and summarize the features of lesions, which is another quantitative problem. For endoscopists who detect lesions while operating, this distraction will undermine their analysis. Therefore, we quantified the change in varices with time and location, relieving endoscopists of the burden of summarizing the features of long varices.

Reporting guidelines for clinical trials involving AI suggested describing how the input data were acquired and selected for the AI intervention. We trained a supportive system to standardize the input images. The system automatically removes poor-quality images guaranteeing the input data are standardized across different trial sites^38^.

The limitations to the current study must be acknowledged. First, in this article, a total of 9.31% of patients were admitted with advanced liver failure (Child‒Pugh C). Because this study was conducted in a tertiary hospital where there are more severe cirrhosis patients. Second, this was a single-arm study rather than a randomized trial, but the performance of ENDOANGEL-GEV and endoscopists were compared. Third, this system did not classify GV according to their location, because the endoscopic location is not the gold standard to determine the supplying vessels and the drainage vessels of GV. Instead, we performed contrast-enhanced computed tomography before treatment.

In conclusion, the present study provided an accurate and interpretable deep learning-based system for the diagnosis and risk stratification of GEV, and our system was validated in a prospective study. The system will increase the effectiveness of interventions tailored to the risk of hemorrhage, thus improving health outcomes and reducing spending on healthcare. This explainable, expert-level system will expand the application scope of AI in quantitative measurement in medicine.

## Methods

### Datasets

#### Datasets and preprocessing

The flowchart of the dataset preparation is shown in Fig. 1. Endoscopic images of GEV used for training, validation, and testing (dataset 1) were collected from Renmin Hospital of Wuhan University, Jingzhou Second People’s Hospital, and Wuhan No. 1 Hospital from January 2nd, 2015, to April 30th, 2019. A doctoral student excluded images with inferior quality (blurs, repetition, or poor preparation). A total of 6034 images from 1156 GEV patients were used to train the models for EV segmentation (model 1), RC segmentation (for both EV and GV, model 2), RC and grade classification (for EV, model 3 and model 4), and GV segmentation (model 5). The size classification model for GV (model 6) has been published^10^. If models segment suspicious varices (or RC) area on an image, the image is classified as varices (RC) positive. If models don’t identify suspicious varices (or RC), the image is classified as varices (or RC) negative. Images from one individual were not split into different datasets.

All images were captured by Olympus (Medical Systems, Tokyo, Japan; GIF-H260Z, CF-HQ290) and Fujifilm systems (Kanagawa, Japan; EC-590WM, EC-600WM). The distribution of images is shown in Supplementary Table 1.

To develop the models, three experts who had more than 10 years of GEV experience (both endoscopists and hepatologists) reviewed all images and classified the images as follows:

EV:

(1) EV/normal esophagus.

(2) RC are graded as 0, 1, 2, or 3 according to their density and distribution: (a) RC0 = absent; (b) RC1 = small in number and localized; (c) RC2 = intermediate between RC1 and RC3; and (d) RC3 = large in number and circumferential.

(3) (a) Grade 1 lesions are straight, small-caliber varices. Small venous dilatations that disappear upon insufflation of the esophagus are not included in this subgroup. (b) Grade 2 lesions are moderately enlarged, beady varices. (c) Grade 3 lesions are markedly enlarged, nodular or tumor shaped varices.

GV:

(1) GV/normal stomach;

(2) RC (0)/RC (1).

(a) RC0 = absent; and (b) RC1 = GV with RC.

(3) Size big (diameter ≥5 mm)/size small (diameter < 5 mm).

All of the above items were classified according to general rules for recording endoscopic findings of GEV^28^. Gold standards were achieved by two or more experts agreed upon results. They will discuss the images which they didn’t reach a consensus on the first classification and finally classified the image into a category. Then, two experts delineated the GEV margins and RC on the images.

#### Training process

Fully convolutional networks (Unet + +) were used to train models 1, 2, and 5 for EV, GV and RC segmentation^29^. Original images were input into framework regardless of resolution, and Unet ++ trained the model in Keras with the labeled maps of experts as the output. Keras is a neural network application programming interface for Python. There was no overlap among the training, validation and test datasets. Cut-off values were chosen to segment the regions of EV, GV, and RC according to the results of the validation datasets. In the later part of the article, the training and validation dataset of Dataset 1 is recorded as Dataset 1(Training and validation dataset), and testing dataset of Dataset 1 is recorded as Dataset 1(Testing dataset).

As guidelines suggest, RC are graded according to their density and distribution^28^. Therefore, RC could be regarded as a group of points, the number and distribution of which were graded. Density-based spatial clustering of applications with noise (DBSCAN) was used to classify the rank of RC (model 3)^39^. DBSCAN was determined by ε and the minimum number of points required to form a dense region (minPts)^39^. Based on ε and minPts, DBSCAN classified the points into core points, reachable points, and outliers. Core points reach n (n ≥ minPts) points within the distance ε. Reachable points could reach core points through a bunch of points directly reachable to each other. If a reachable point cannot reach more than minPts points, it is the cluster’s edge.

All results were compared with the gold standards, retaining the best model with minPts as 1 and ε as math.sqrt(w*h/6.5).

Model 4 and model 6 were deep learning convolutional neural networks trained based on Fast.ai to classify the results of model 1 and model 5.

#### Supportive models

Supportive model 1 removed the unqualified images, including images with blurring, digital chromo, biopsy forceps, and flushing water. 38,422 endoscopic images were classified into 15,084 qualified images and 23,338 unqualified images (blurry, digital chromo, biopsy forceps, duplicate, flushing water) by doctoral students to develop supportive model 1. A deep convolutional neural network was trained based on ResNet 50.

Supportive model 2 was used to classify the esophagogastroduodenoscopy images into 26 sites and retain images in the esophagus, squamocolumnar junction, fundus lesser curvature, fundus anterior wall, fundus greater curvature, and fundus posterior wall^14,40^.

Supportive model 3 was to remove the images with inadequate inflation. Images in inadequate inflation section or inadequate inflation caused by breath will be removed by supportive model 3. It was trained using 3813 inadequate inflation images and 6392 adequate inflations based on ResNet 50.

#### Training devices

1. Hardware parameters: All models were trained on Windows 10 Professional operating system. CPU versions are Intel® Core™ i7-8700@3.20Ghz and @3.19 GHz. GPU is NVIDIA GeForce RTX 2080 (memory size: 8 GB, memory bandwidth: 256 bits, frequency: 7000 MHz).
2. Software Environment: Programming language is Python 3.6.5. Deep Learning Frameworks are TensorFlow 1.12.2 and Keras 2.2.5.
3. Python packages: OpenCV-python 4.5.3.56, NumPy 1.19.5, and Pandas 1.1.5.

#### Validation dataset (dataset 2)

To validate the ability of the system to diagnose and classify risk factors in real-time, ENDOANGEL-GEV was tested using sequential images clipped from 141 esophagogastroduodenoscopy videos (25 frames per second) from 3 independent cohorts (Wuhan Puren Hospital, Central Hospital of Enshi Tujia and Miao Autonomous Prefecture and Renmin Hospital of Wuhan University). Experts affiliated with the hospitals established the gold standard. Smoothing was used by taking the results of three or more images out of five consecutive qualified images as the prediction result.

#### Prospective study (dataset 3)

The system was installed on computers in the endoscopy unit of Renmin Hospital of Wuhan University, and endoscopic videos (7 frames per second) of prospective cirrhotic patients were analyzed to validate the system in the clinic. Endoscopists were blinded to the results of the system. The gold standards were the same as those in the training dataset. Three supportive models were added to the system and activated in order to process the videos.

#### Patients

This prospective observational study was conducted at Renmin Hospital of Wuhan University from July 1st, 2020, to April 30th, 2021. Cirrhotic patients presented to Renmin Hospital of Wuhan University were invited to participate in this study. The inclusion criteria were as follows: (1) cirrhosis diagnosed by histology or by both blood samples and two methods of imaging, ultrasound and computed tomography/magnetic resonance imaging; (2) age between 18 and 80 years; and (3) no previous EV or GV bleeding and never received endoscopic treatment, surgical treatment, or transjugular intrahepatic portosystemic shunt for EV or GV before. The exclusion criteria included (1) gastrointestinal malignancies before participation; (2) a history of esophagus or stomach surgery; (3) severe diseases of other organs or infections with a prehepatic or posthepatic origin; and (4) refusal to give informed consent to participate in the study.

Data on the presence or absence of ascites, jaundice, and hepatic encephalopathy were collected before endoscopy. A blood sample under fasting conditions was taken before endoscopy to assess liver disease etiology and severity (Child‒Pugh score).

#### Endoscopy

All eligible patients underwent endoscopy, performed using CF-HQ290, CF-Q260AI (Olympus Optical, Tokyo, Japan) EC-590WM, or EC-600WM systems (Fujifilm, Kanagawa, Japan). The endoscopists were six staff members of the Gastroenterology Department in Renmin Hospital of Wuhan University, Wuhan, China, with an endoscopic experience of 6.67 ± 2.58 years.

EV and GV were classified and recorded according to the general rules of the Japan Research Society for Portal Hypertension. All patients were treated by the endoscopists mentioned above according to the latest guidelines^2^. The indication for primary prophylaxis was small varices (grade 1) with Child‒Pugh C, or the presence of medium (grade 2) to large varices (grade 3) with or without RC on varices. All patients were followed up for at least 6 months. Adverse events were considered one of the following complications: upper gastrointestinal hemorrhage from variceal bleeding confirmed by endoscopy and death.

The study was carried out in compliance with the Declaration of Helsinki. The study protocol was approved by the ethics committees of the Renmin Hospital of Wuhan University (Reference number: 2019K-K094(Y01)). Written informed consent was obtained from all prospective patients. The ethics committee waived the requirement of informed consent for retrospectively collected information.

#### A questionnaire on the satisfaction of ENDOANGEL-GEV

Five endoscopists were asked to watch three videos applied by ENDOANGEL-GEV and ENDOANGEL (previously published)^10^. They filled in a questionnaire after watching the videos. The questionnaire contains three questions on the two systems’ accuracy, helpfulness, and trustworthiness. They ranked five levels of agreement: strongly agree, agree, neutral, disagree, and strongly disagree.

#### Outcomes

The primary outcome of the study was the accuracy of ENDOANGEL-GEV in detecting GEV on dataset 3(Prospective study). The secondary outcomes were the metrics of ENDOANGEL-GEV and endoscopists to detect and rank risk factors for GEV, the comparison results between ENDOANGEL-GEV and endoscopists, and the diagnostic value of six endoscopists for detecting risk factors and risk stratification.

#### Sample size

We assumed ENDOANGEL-GEV could reach the diagnostic accuracy of 90% in a single-arm group study with objective performance criteria. With a power of 90%, a two-sided significance level of 0.05, 158 patients were required. Assuming a drop-out rate of 5%, the target sample size was 166. The sample size was calculated using Power Analysis and Sample Size 15.

#### Statistical analysis

Precision, recall, and Intersection over union (IoU) were calculated to assess the segmentation.

IoU was defined as the relative overlap between the predicted bounding box and the ground-truth bounding box.

Precision = True positive area/(True positive area + False positive area)

Recall = True positive area/(True positive area + False negative area)

Accuracy, sensitivity, specificity, positive predictive value, and negative predictive value were calculated. Categorical variables were compared by using the chi-square test (McNemar test). *P* values < 0.05 were considered statistically significant. All calculations were performed using SPSS 23 (IBM, Chicago, Illinois, USA).

### Reporting summary

Further information on research design is available in the Nature Research Reporting Summary linked to this article.

## Supplementary information

Supplementary materials

video 1

Reporting Summary

## Data Availability

Individual de-identified data and pretraining model, and source code reported in this article will be shared for investigators 12 months after article publication. Data requesters could contact the corresponding author to gain access. The codes are uploaded on Github. (https://github.com/endo-angel/gastroesophageal-varices).
