# Supplementary material for: An interpretable artificial intelligence system for detecting risk factors of gastroesophageal variceal bleeding
Source: NPJ Digit Med. 2022 Dec 19;5:183. doi: 10.1038/s41746-022-00729-z (PMC9763258; doi:10.1038/s41746-022-00729-z)
Supplement: Supplementary file 1 — Supplementary materials [file 41746_2022_729_MOESM1_ESM.pdf]

Supplementary materials

Tables

Supplementary Table 1. Distribution of images/videos from different suppliers/institutions among three datasets

	**Training and validation dataset**	**Dataset 1 (Testing dataset)**	**Dataset 2 (Validation dataset)**	**Dataset 3（Prospective study）**
**Institutions**				
Renmin Hospital of Wuhan University	3339	1050	2853	161
Wuhan NO.1 Hospital	1023	-	-	-
Jingzhou second people's Hospital	622	-	-	-
Wuhan Puren Hospital	-	-	3268	-
Central Hospital of Enshi Tujia and Miao Autonomous Prefecture	-	-	4888	-
**Suppliers**				
Olympus	3988	743	7146	96
Fujifilm	996	307	3863	65

Supplementary table 2. Accuracy of model 4 (grade classification model) on three datasets

	**Grade 1, %(95% CI)**	**Grade 2, %(95% CI)**	**Grade 3, %(95% CI)**
**Dataset 1(Testing dataset)**	90.00(83.05, 94.68)	93.19(89.87, 95.68)	98.27(90.76, 99.96)
**Dataset 2(Validation dataset)**	94.84(91.62, 96.04)	93.67(90.57, 94.75)	93.88(89.74, 96.82)
**Dataset 3（Prospective study）**	95.45(77.16, 99.88)	95.24(88.25, 99.19)	87.50(67.64, 98.54)

Supplementary table 3. Performance of model 3(RC classification model) on three datasets

	RC(0), %(95 % CI)	RC(1), %(95 % CI)	RC(2), %(95 % CI)	RC(3), %(95 % CI)	Sensitivity, %(95 % CI)	Specificity, %(95% CI)
**EV**						
Dataset 1(Testing dataset)	96.92(93.48, 98.64)	89.24(80.68, 94.44)	91.67(83.04, 96.30)	95.83(89.07, 98.65)	98.53(96.04, 99.53)	96.92(93.48, 98.64)
Dataset 2(Validation dataset)	92.54(91.23, 93.67)	97.60(96.05, 98.57)	91.52(88.54, 93.81)	92.59(88.35, 95.43)	100.00(99.65, 100.00)	92.54(91.23, 93.67)
Dataset 3（Prospective study）	94.29(85.27, 98.15)	90.63(73.83, 9754)	100(78.12, 100.00)	100(62.88, 100.00)	95.00(85.18, 98.70)	94.29(85.27, 98.15)
**GV**						
Dataset 1(Testing dataset)	-	-	-	-	90.03(86.09, 92.98)	91.70(87.15, 94.80)
Dataset 2(Validation dataset)	-	-	-	-	91.58(90.80, 92.30)	91.10(89.85, 92.20)
Dataset 3（Prospective study）	-	-	-	-	96.15(89.87, 98.76)	85.71(56.15, 97.48

EV, esophageal varices; GV, gastric varices; RC, red color sign.

Supplementary table 4. Metrics of ENDOANGEL-GEV and endoscopists for the detection of EV on Dataset 3（Prospective study）

**The number of images**		**ENDOANGEL-GEV**		**Endoscopists**	
		Normal	EV	Normal	EV
**Gold standard**	Normal	28	2	29	1
	EV	0	131	1	130
**Diagnostic yield (95% CI), %**					
Accuracy		98.76(95.30-99.95)		98.76(95.30-99.95)	
Sensitivity		100.00(96.44-100.00)		99.23(95.19-99.96)	
Specificity		93.33(76.49-98.83)		96.66(80.94-99.82)	
Positive predictive value		98.49(94.12-99.73)		99.23(95.19-99.96)	
Negative predictive value		100.00(94.12-99.73		96.66(80.94-99.82)	

EV, esophageal varices.

Supplementary table 5. Metrics of ENDOANGEL-GEV and endoscopists for GV detection on Dataset 3（Prospective study）

The number of images		ENDOANGEL-GEV		Endoscopists	
		Normal	GV	Normal	GV
**Gold standard**	Normal	38	3	41	0
	GV	1	119	2	118
**Diagnostic yield (95% CI), %**					
Accuracy		97.52(93.57-99.25)		98.76(95.30-99.95)	
Sensitivity		99.16(94.76-99.95)		98.33(9.50-99.71)	
Specificity		92.68(78.99-98.09)		100.00(89.33-100.00)	
Positive predictive value		97.54(92.44-99.36)		100.00(96.07-100.00)	
Negative predictive value		97.43(84.92-99.86)		95.34(82.94-99.19)	

GV, gastric varices.

Supplementary table 6. Metrics of ENDOANGEL-GEV and endoscopists for detecting and classifying RC on Dataset 3（Prospective study）

The number of EV images		ENDOANGEL-GEV				Endoscopists			
		RC(0)	RC(1)	RC(2)	RC(3)	RC(0)	RC(1)	RC(2)	RC(3)
	RC(0)	66	3	1	0	40	30	0	0
**Gold**	RC(1)	3	30	0	0	7	26	0	0
**standard**	RC(2)	0	0	18	0	1	3	13	1
	RC(3)	0	0	0	9	0	0	1	8
**Diagnostic yield (95% CI),%**									
Accuracy		94.62, (89.11- 97.56) [1]				66.92, (58.44- 74.44)			

RC, red color sign; EV, esophageal varices. [1], $p < 0.001$.

Supplementary table 7. Metrics of ENDOANGEL-GEV and endoscopists for the detection of RC(GV) on Dataset 3（Prospective study）

The number of GV images		ENDOANGEL-GEV		Endoscopists	
		RC (+)	RC (-)	RC (+)	RC (-)
**Gold standard**	RC (+)	12	2	7	7
	RC (-)	4	100	29	75
**Diagnostic yield (95% CI), %**					
Accuracy		94.92(89.26-98.11)[1]		69.49(60.34-77.63)	
Sensitivity		85.71(56.15-97.48)		50.00(24.04-75.95)	
Specificity		96.15(89.87-98.76)		72.11(62.32-80.24)	
Positive predictive value		75.00(47.40-91.66)		19.44(8.80-36.56)	
Negative predictive value		98.03(92.40-99.65)		91.46(82.65-96.20)	

RC, red color sign; GV, gastric varices. [1], $p < 0.001$.

Supplementary table 8. Metrics of ENDOANGEL-GEV and endoscopists for the classification of grade (EV) on Dataset 3（Prospective study）

The number of EV images		ENDOANGEL-GEV			Endoscopists		
		Grade 1	Grade 2	Grade 3	Grade 1	Grade 2	Grade 3
**Gold standard**	Grade 1	21	1	0	18	4	0
	Grade 2	2	80	2	8	59	17
	Grade 3	0	3	21	0	3	21
**Diagnostic yield (95% CI), %**							
Accuracy		94.57(89.14-99.90) [1]			75.97(67.88-82.56)		

EV, esophageal varices. [1], $p < 0.001$.

Supplementary table 9. Metrics of ENDOANGEL-GEV and endoscopists for the classification of size (GV) on Dataset 3（Prospective study）

The number of GV images		ENDOANGEL-GEV		Endoscopists	
		Small	Big	Small	Big
**Gold standard**	Small	80	2	76	6
	Big	2	34	6	30
Accuracy		96.61(91.32-98.96)		89.83(82.93-94.22)	
Sensitivity		94.44(79.98-99.03)		83.33(66.52-93.03)	
Specificity		97.56(90.64-99.57)		92.68(84.17-96.99)	
Positive predictive value		94.44(79.98-99.03)		83.33(66.52-93.03)	
Negative predictive value		97.56(90.64-99.57)		92.68(84.17-96.99)	

GV, gastric varices.

Supplementary table 10. The accuracy of six endoscopists for detecting risk factors and risk stratification on Dataset 3（Prospective study）

	The accuracy for classifying grade of EV, % (95% CI)	The accuracy for classifying RC of EV, % (95% CI)	The accuracy for classifying size of GV, % (95% CI)	The accuracy for classifying RC of GV, % (95% CI)	The accuracy for risk stratification of EV, % (95% CI)	The accuracy for risk stratification of GV, % (95% CI)
**Endoscopist A**	89.47(67.37-98.30)	78.95(56.11-92.05)	83.33(59.95-94.99)	72.22(48.80-87.83)	84.21(61.60-95.32)	72.22(48.80-87.83)
**Endoscopist B**	96.00(78.86-99.99)	68.00(48.27-82.94)	95.24(75.58-99.99)	80.95(59.41-92.92)	84.62(65.85-94.47)	90.48(69.88-98.55)
**Endoscopist C**	93.10(76.97-99.15)	72.41(54.09-85.50)	92.00(73.90-98.91)	56.00(7.05-73.35)	89.66(72.81-97.22)	88.00(69.21-96.67)
**Endoscopist D**	95.65(77.34-99.99)	56.52(36.69-74.39)	82.61(62.26-93.63)	56.52(36.79-74.39)	90.91(71.00-8.66)	73.91(53.24-87.74)
**Endoscopist E**	76.19(54.51-89.77)	71.43(49.79-86.44)	94.97(73.52-99.99)	68.42(45.80-84.84)	76.19(54.51-89.77)	78.95(56.11-92.05)
**Endoscopist F**	91.67(62.47-99.99)	58.33(31.89-80.74)	91.67(62.47-99.99)	100.00(71.80-100.00)	83.33(54.00-96.50)	91.67(62.47-99.99)

Supplementary table 11. Accuracy of the system for identifying six varices relevant anatomical landmarks on Dataset 3（Prospective study）

	The number of images	Accuracy, %(95% CI)
**Esophagus**	121152	99.27(99.22-99.31)
**Squamocolumnar junction**	8050	97.64(97.27-97.95)
**Fundus(G)**	46073	98.34(98.21-98.45)
**Fundus(P)**	40676	96.37(96.18-96.54)
**Fundus(A)**	46994	94.88(94.67-95.07)
**Fundus(L)**	14860	89.68(89.17-90.15)

Supplementary table 12. mIoU of the system on images from Olympus and Fujifilm on Dataset 1(Testing dataset)

	Olympus, mIoU(95% CI)	Fujifilm, mIoU(95% CI)	P value
**Model 1(EV segmentation model)**	0.8089(0.6884, 0.9294)	0.8086(0.7005, 0.9167)	0.984
**Model 5(GV segmentation model)**	0.8145(0.6619, 0.9581)	0.8118(0.6275, 0.9961)	0.927

mIoU, mean intersection over union; EV, esophageal varices; GV, gastric varices;.

Supplementary table 13. Accuracy of the system on images from Olympus and Fujifilm on Dataset 1(Testing dataset)

	Olympus, %(95% CI)	Fujifilm, %(95% CI)	P value
**Model 3(RC classification model)**	93.51(90.77, 95.49)	95.83(92.68, 97.72)	0.321
**Model 4(grade classification model)**	90.64(88.22, 92.62)	91.30(83.85, 95.64)	0.899
**Model 6(Size classification model for GV)**	91.88(88.92, 94.12)	85.51(77.08, 91.26)	0.109

RC, red color sign; GV, gastric varices.

Supplementary table 14. Characteristics of patients suffered bleeding during follow up

	Bleeding site	Child-Pugh	ENDOANGEL-GEV		Endoscopist	
			**RC**	**Grade/ Size**	**RC**	**Grade/ Size**
**Case 1**	Esophagus	B	RC 3	Grade 3	RC 3	Grade 3
**Case 2**	Esophagus	B	RC 2	Grade 2	RC 2	Grade 2
**Case 3**	Esophagus	C	RC 0	Grade 2	RC 0	Grade 2
**Case 4**	Fundus	A	RC 0	Size 0	RC 1	Size 0
**Case 5**	Fundus	B	RC 0	Size 1	RC 1	Size 0
**Case 6**	Fundus	C	RC 0	Size 1	RC 0	Size 1

GEV, gastroesophageal varices; RC, red color sign.

Figure legends

Supplementary figure 1. Representative unqualified images. A. unqualified images. B. inadequate inflation images.

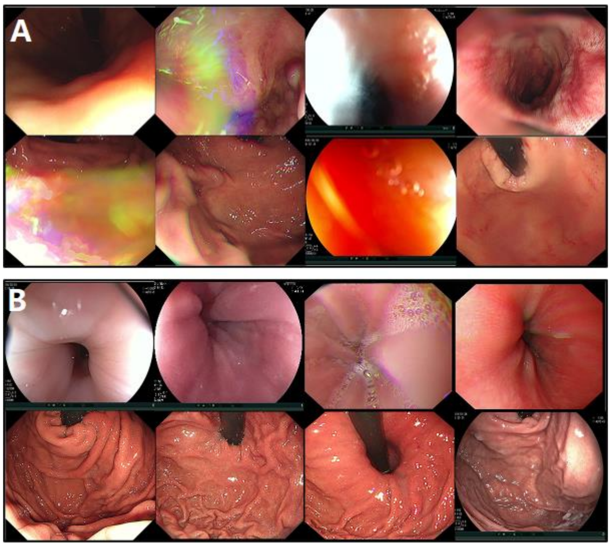

Supplementary figure 2. Representative original images and qualified images filtered by supportive models. A. original images. B. qualified images.

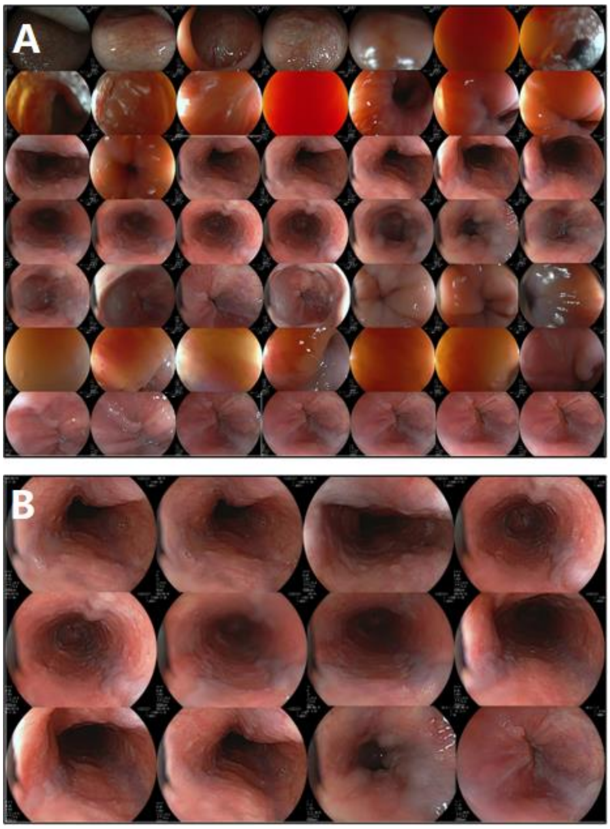

Supplementary figure 3. Typical wrong images and wrong regions of ENDOANGEL-GEV and

endoscopists for detecting RC. The blue box refers to ENDOANGEL-GEV, and the red box refers to endoscopists. The blue box is slightly larger than the enclosing rectangle of the region predicted by model in order to observe the boundary of RC.

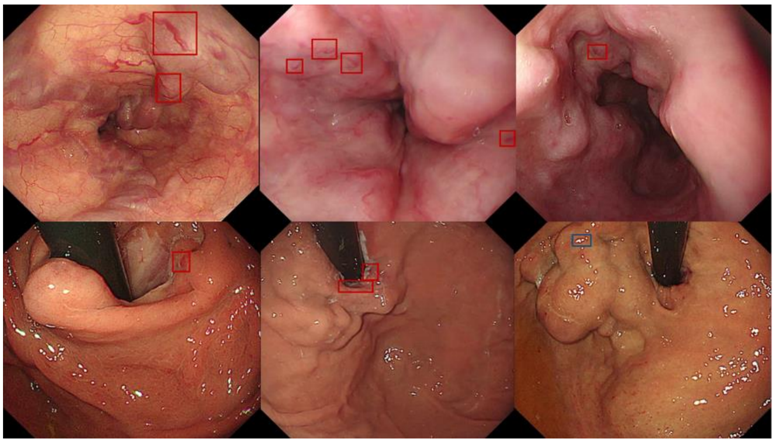

RC, red color sign.

Supplementary figure 4. Level of agreement with Likert scale questions about comparing ENDOANGEL-GEV and ENDOANGEL (previously published) .

Question 1. ENDOANGEL-GEV is more accurate than ENDOANGEL.

Question 2. ENDOANGEL-GEV is more trust worthy than ENDOANGEL.

Question 3. ENDOANGEL-GEV is more helpful than ENDOANGEL.

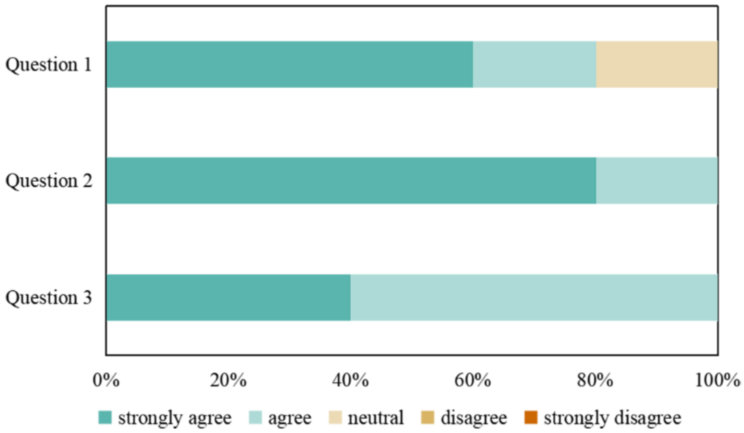

Supplementary Video 1. A representative video shows how the system detects varices and risk factors
