# Supplementary material for: An interpretable artificial intelligence system for detecting risk factors of gastroesophageal variceal bleeding
Source: NPJ Digit Med. 2022 Dec 19;5:183. doi: 10.1038/s41746-022-00729-z (PMC9763258; doi:10.1038/s41746-022-00729-z)
Supplement: Supplementary file 3 — Reporting Summary [file 41746_2022_729_MOESM3_ESM.pdf]

nature portfolio

Corresponding author(s): Honggang Yu

Last updated by author(s): Jun 4, 2022

Reporting Summary

Nature Portfolio wishes to improve the reproducibility of the work that we publish. This form provides structure for consistency and transparency in reporting. For further information on Nature Portfolio policies, see our Editorial Policies and the Editorial Policy Checklist.

Statistics

For all statistical analyses, confirm that the following items are present in the figure legend, table legend, main text, or Methods section.

n/a	Confirmed	
☐	☒	The exact sample size (*n*) for each experimental group/condition, given as a discrete number and unit of measurement
☐	☒	A statement on whether measurements were taken from distinct samples or whether the same sample was measured repeatedly
☐	☒	The statistical test(s) used AND whether they are one- or two-sided<br>*Only common tests should be described solely by name; describe more complex techniques in the Methods section.*
☒	☐	A description of all covariates tested
☒	☐	A description of any assumptions or corrections, such as tests of normality and adjustment for multiple comparisons
☐	☒	A full description of the statistical parameters including central tendency (e.g. means) or other basic estimates (e.g. regression coefficient) AND variation (e.g. standard deviation) or associated estimates of uncertainty (e.g. confidence intervals)
☐	☒	For null hypothesis testing, the test statistic (e.g. *F*, *t*, *r*) with confidence intervals, effect sizes, degrees of freedom and *P* value noted<br>*Give P values as exact values whenever suitable.*
☒	☐	For Bayesian analysis, information on the choice of priors and Markov chain Monte Carlo settings
☒	☐	For hierarchical and complex designs, identification of the appropriate level for tests and full reporting of outcomes
☐	☒	Estimates of effect sizes (e.g. Cohen's *d*, Pearson's *r*), indicating how they were calculated

Our web collection on statistics for biologists contains articles on many of the points above.

Software and code

Policy information about availability of computer code

Data collection	No software was used
Data analysis	Precision, recall, and Intersection over union (IoU) were calculated to assess the segmentation. IoU was defined as the relative overlap between the predicted bounding box and the ground-truth bounding box.<br>Precision = True positive area / (True positive area + False positive area)<br>Recall = True positive area / (True positive area + False negative area)<br>Accuracy, sensitivity, specificity, positive predictive value, and negative predictive value were calculated. Categorical variables were compared by using the chi-square test (McNemar test). P-values< 0.05 were considered statistically significant. All calculations were performed using SPSS 23 (IBM, Chicago, Illinois, USA).

For manuscripts utilizing custom algorithms or software that are central to the research but not yet described in published literature, software must be made available to editors and reviewers. We strongly encourage code deposition in a community repository (e.g. GitHub). See the Nature Portfolio guidelines for submitting code & software for further information.

Data

Policy information about availability of data

All manuscripts must include a data availability statement. This statement should provide the following information, where applicable:

- Accession codes, unique identifiers, or web links for publicly available datasets
- A description of any restrictions on data availability
- For clinical datasets or third party data, please ensure that the statement adheres to our policy

Individual de-identified participant data and pretraining model, software, and source code reported in this article will be shared for investigators after article publication. Data requesters could contact the corresponding author to gain access.

Human research participants

Policy information about studies involving human research participants and Sex and Gender in Research.

Reporting on sex and gender	In prospective observational study, 161 patients were analyzed in this research (117 men, 44 women; mean age 57.41 years, range 32-79 years).
Population characteristics	6034 images from 1156 GEV patients were used to train the models for esophageal varices (EV) segmentation (model 1), RC segmentation (for both EV and gastric varices (GV), model 2), RC and form classification (for EV, model 3 and model 4)GV segmentation (model 5). Size classification model for GV (model 6) has been published.9 Images from one individual were not split into different datasets.
Recruitment	Endoscopic images of GEV used for training, validation, and test (dataset 1) were from Renmin hospital of Wuhan University, Jingzhou second people's Hospital, and Wuhan No. 1 Hospital, from January 2nd, 2015, to April 30th, 2019.
Ethics oversight	The study was carried out in compliance with the Declaration of Helsinki. The study protocol was approved by the ethics committees of the Renmin Hospital of Wuhan University (Reference number2019K-K094(Y01)). Informed written consent was obtained from all prospective patients. The ethics committee waived the requirement of informed consent for retrospectively collected information.

Note that full information on the approval of the study protocol must also be provided in the manuscript.

Field-specific reporting

Please select the one below that is the best fit for your research. If you are not sure, read the appropriate sections before making your selection.

☒ Life sciences ☐ Behavioural & social sciences ☐ Ecological, evolutionary & environmental sciences

For a reference copy of the document with all sections, see nature.com/documents/nr-reporting-summary-flat.pdf

Life sciences study design

All studies must disclose on these points even when the disclosure is negative.

Sample size	We assumed ENDOANGEL-GEV could reach the diagnostic accuracy of 90% in a single-arm group study with objective performance criteria. With a power of 90%, a two-sided significance level of 0.05, and a superiority margin of 0.05, 158 patients were required. Assuming a drop-out rate of 5%, the target sample size was 166. The sample size was calculated using Power Analysis and Sample Size 15.
Data exclusions	The exclusion criteria include(1) gastrointestinal malignancies before participation; (2) a history of esophagus or stomach surgery; (3) severe diseases of other organs or infections with a prehepatic or posthepatic origin; (4) refusal to give informed consent to participate in the study.
Replication	To develop models, three experts who have experience of GEV for more than 10 years (They were both endoscopists and hepatologists.) reviewed all images and classified the images into:<br>EV:<br>(1) EV/ normal esophagus;<br>(2) RC (0)/RC (1)/RC (2)/ RC (3);<br>RC are graded as 0, 1, 2, or 3 according to their density and distribution: (a) RC0 = absent; (b) RC1 = small in number and localized; (c) RC2 = intermediate between RC1 and RC3; and (d) RC3 = large in number and circumferential.<br>(3) Form 1/form2/form 3.<br>(a) F1 lesions are straight, small-caliber varices. Small venous dilatations that disappear on insufflation of the esophagus are not included in this subgroup; (b) F2 lesions are moderately enlarged, beady varices; (c) F3 lesions are markedly enlarged, nodular or tumor shaped varices.<br>GV:<br>(1) GV/ normal stomach;<br>(2) RC (0)/RC (1);<br>(a) RC0 = absent; (b) RC1 = GV with RC.

	(3) Size big(GV: diameter ≥5mm)/size small(GV: diameter?5mm).<br>All above items were classified according to general rules for recording endoscopic findings of GEV<br>Fully convolutional networks (Unet++) were used to train models 1, model 2, and model 5 for EV, GV and RC segmentation.24 Original images were input into framework regardless of resolution, and Unet++ trained the model in Keras with labeled maps of experts as output. Cut-off values were chosen to segment the regions of EV, GV, and RC according to the result of validation datasets.<br>As guidelines suggest, RC are graded according to their density and distribution.22 So RC could be regarded as a group of points, the number and distribution of which were graded. Density-based spatial clustering of applications with noise (DBSCAN) was used to classify the rank of RC(Model 3).34 All results were compared with gold standards, retaining the best model with minPts is 1 and ε is math.sqrt(w*h/6.5).<br>Model 4 and model 6 was deep learning convolutional neural networks trained based on Fast.ai to classify the result of model 1 and model 5.
Randomization	Images were randomly spilt at an 8:1:1 ratio for training, validation and testing of the models. There is no overlap among training, validation and test datasets. Cut-off values were chosen to segment the regions of EV, GV, and RC according to the result of validation datasets.
Blinding	System was installed on the computer in the endoscopy unit of Renmin Hospital of Wuhan University and analyzed endoscopic videos (25 frames per second) of prospective cirrhotic patients to validate the system in the clinic. Endoscopists were blind to the results of system.

Reporting for specific materials, systems and methods

We require information from authors about some types of materials, experimental systems and methods used in many studies. Here, indicate whether each material, system or method listed is relevant to your study. If you are not sure if a list item applies to your research, read the appropriate section before selecting a response.

Materials & experimental systems

n/a	Involved in the study	
☒	☐	Antibodies
☒	☐	Eukaryotic cell lines
☒	☐	Palaeontology and archaeology
☒	☐	Animals and other organisms
☒	☐	Clinical data
☒	☐	Dual use research of concern

Methods

n/a	Involved in the study	
☒	☐	ChIP-seq
☒	☐	Flow cytometry
☒	☐	MRI-based neuroimaging
